# Coming To Our Census: How Social Statistics Underpin Our Democracy (And Republic)

**DOI:** 10.1162/99608f92.c871f9e0

**Published:** 2020-01-31

**Authors:** Teresa A. Sullivan

**Affiliations:** 1 Interim Provost and Executive Vice President for Academic Affairs, Michigan State University, and President Emerita and University Professor, University of Virginia

**Keywords:** census, democracy, statistical infrastructure, data errors, data privacy

## Abstract

The 2020 Census provides the opportunity to reflect on the key role statisticians, demographers, and other social scientists play in safeguarding American democracy. Democracy requires numbers for its proper functioning, and there is now a large statistical infrastructure of which the constitutionally mandated census is the keystone. Mistrust of the government is a major obstacle for the census, potentially affecting both accuracy and completeness. The mistrust is stimulated by fears of individual or household census data being willingly or inadvertently shared with other government agencies (data privacy issues) or even foreign actors (hacking). As two 2019 Supreme Court decisions in juxtaposition suggest, no checks or balances protect the integrity of the census. The professional integrity of statisticians is the best defense of the census.

Throughout the United States, thousands of people are preparing to conduct America’s largest and most expensive data collection project: the 2020 Census. The census will be our decennial family portrait, with myriad uses in both the public and private sectors. Sometimes underplayed, however, is the role of the census in undergirding our democracy and republic through elected representation and fair allocation of resources.

## 1. Democracy Requires Numbers for Its Proper Functioning

Democracy has always needed numbers, if only to determine the size needed for a majority. Beyond that bare minimum, however, the numbers are needed to ensure the civic empowerment of each individual. The ideal relationship of the individual to the state was described in the Declaration of Independence as “all men are created equal.” To be sure, the Founders’ sense of equality was limited to free White men and preferably White men who were landholders; over time our ideas of equality and fairness have expanded to include women and people of color, as well as the landless. Even in the beginning, however, each inhabitant—whether or not enfranchised—was to be enumerated apart from “Indians not taxed.”^1^

While numerical equality is necessary for full participation in the democracy, it is not sufficient. Because members of Congress and legislatures represent geographic areas, individuals must also be counted within their geographic areas. Geographic areas rely critically on census numbers for two functions: representation to decision-making bodies and allocation of resources back to geographic areas (*Wisconsin v. City of New York*, 1966, pp. 5-6).

Consider, for a moment, a nation that has no statistics or that disregards what statistics it has. How are decisions made? Without a sense of proportionality, the functions of government will be described with terms such as ‘rotten boroughs’ and ‘pork barrels.’ It will be very hard to satisfy anyone that the decisions were ‘fair.’ Especially in a nation as politically divided as the United States is today, believing that all decisions are based merely on partisan politics is toxic.

## 2. There Is a Social Statistics Infrastructure

For its first half-century, the United States was divided over whether government should play a role in building infrastructure and, if so, what role it should play (Larson, 2001). Gradually the privately owned turnpikes became a network of public roads and bridges. Each successive technological innovation generated its own controversies over the proper role of government—first canals and river navigation, then harbors, railroads, pipelines, airports, and now Internet access and functioning (Blevins, 2019). Notwithstanding all the arguments about degree and kind, and who shall pay for what, it is well established that the government plays a role in physical infrastructure. Physical infrastructure is now used as an example of a public good. The current infrastructure debate in Washington is not about whether physical infrastructure should be publicly funded, but rather about the level of funding for maintaining and expanding physical infrastructure.

Although not always as explicitly recognized, there is today a parallel to physical infrastructure in a social statistical infrastructure, composed of many types of indicators. Examples include economic forecasts, weather warnings, and seismic tracking. Myriad other government functions depend crucially upon the generation of actionable data. As with physical infrastructure, conceptually these functions *could* be privatized—people could subscribe to their own econometrician, or get a private weather service, or use a university’s seismograph. For most Americans, however, using government data has become so routine as to be unnoticed—much as the fish doesn’t notice the water. And because these data often describe things that are intangible or invisible—the money supply, the jet stream, tectonic plates—we do not recognize the statistics as an infrastructure in the same way as a bridge or an airport control tower. Nevertheless, the statistics underpin entire industries. Both the public and private sectors use these data for planning, for marketing, and for human welfare. The fish will notice the lack of water when its pond dries up, and Americans would notice the absence of their statistical infrastructure.

## 3. The U.S. Census Is the Cornerstone of the Social Statistical Infrastructure

The decennial U.S. census is the source for the numbers democracy needs, and because of its constitutional mandate, it serves as the cornerstone of the social statistical infrastructure. The democratic functions of the census could be described broadly as representation and allocation.

### 3.1 Representation and Allocation

It seems fair that the government would treat similarly situated individuals in the same way. In a republic, where representatives are elected by direct vote, having districts of approximately equal population size seems fair, and in the United States was imposed by the Supreme Court in its requirement for one-person-one-vote (Baker v. Carr, 1962; Reynolds v. Sims, 1964; Wesberry v. Sanders 1964).

Our democratic republic provides people with many goods and services. The funding is substantial: up to $880 billion estimated over the decade that the census data are used. Fairness in this distribution is important. Not many Americans would be happy with a system that provided Medicare benefits only for New York but not for Illinois, or that provided Social Security only for people living in rural areas but not cities. Some services are shared by all, such as highways, and others are distributed to individuals based on their characteristics: age (such as education or Medicare), veteran status, health needs, income level, and much more. But whether the goods are received in common or distributed, numbers help determine what is fair.

By linking individuals to their geographic location, the census provides the basis for representation. By providing information on age, sex, household size, race, and ethnicity, the census provides information on the demand for common public goods such as highways and utilities, and on the demand for distributed goods such as education and medical care. Census data are the first source for denominators for measuring processes as disparate as the per capita consumption of water in the Southwest, the incidence of measles in a state, and the rates of net increase or decrease in rural populations. The census provides the basis for sampling frames for surveys in both public and private sectors. Free, valid public data is itself a public good.

Democracy needs the social statistical infrastructure and the census has emerged as the cornerstone of that social infrastructure. This is because, uniquely among major data sources, the census is mandated by the Constitution.

## 4. The Census Is Constitutionally Required

Based on their understanding of the Jewish scriptures, many Europeans in the early modern era were superstitious about counting the population.^2^ Their superstitions and their political apprehensions were mutually reinforcing: the monarch’s reasons for enumeration were collecting taxes and calculating military might. Mistrust of government engendered mistrust of efforts to enumerate the population.

Despite such misgivings, the young American republic needed a census. Its elected House of Representatives would be apportioned by the population of each state. The U.S. Constitution in Article 1, Section 2, as amended by section 2 of the Fourteenth Amendment states: *“Representatives shall be apportioned among the several States according to their respective numbers, counting the whole number of persons in each State, excluding Indians not taxed.”*

There was an obvious moral hazard in letting each state do its own count, and so a federally run census was created in that same section: *“The actual Enumeration shall be made within three Years after the first Meeting of the Congress of the United States, and within every subsequent Term of ten Years, in such Manner as they [Congress] shall by Law direct.”*

The Constitution thus sets forth the why, the who, the when, and the where of the census, and it assigned the how to Congress. Early members of Congress were very active in this process. James Madison is said to have contributed most of the questions in the first enumeration in 1790, and Thomas Jefferson as secretary of state led the first census.^3^ Later, Congresses played an active role in planning the enumeration. By 1902 there was a permanent census bureau, with most operational authority delegated to the secretary of commerce by 1929. Then and now, Congress has passed statutes to provide direction for the census. To give a few current examples, the secretary must report regularly to Congress about the census, both houses may hold hearings concerning the census, and any appropriations must originate in the House. The secretary of commerce and the census director are political appointees and must be confirmed by the Senate.

## 5. How Mistrust of Government Threatens the Quality of Census Data

Mistrust of census-taking did not end with terminating monarchical control in America.^4^ There have always been people who would like to avoid official record-keeping because of illegal activity or their own deep suspicions. Some people who value personal privacy fear that the Census Bureau will violate their privacy through intentional sharing of their information or linking their records to other data bases. A mirror-image of this concern is the belief that all the information is already linked so the census is not necessary. Additional sources of mistrust include public commentaries that cast doubt on data (‘fake news’), offer substitute data (‘alternative facts’), or impugn the motives of the government personnel who produce the data (‘deep state’). Finally, there is the possibility that the bureau’s own efforts to be honest about errors in the data may fuel further mistrust of the census enterprise.

Two of the touchstone criteria for a good census have always been accuracy of the content and completeness of the count. Protection of privacy is an important additional criterion. In the digital era, cybersecurity needs to be added to the list, not only for bolstering the protection of privacy but also for preventing mischief that undermines accuracy and completeness. Mistrust of the government’s conduct of the census and subsequent use of the data can compromise each of these criteria.

## 6. The Citizenship Question

The public discussion of the secretary of commerce’s effort to add a citizenship question to the 2020 census illustrates how government action can intensify public mistrust. The recent 92-page decision of the Supreme Court in the case of the citizenship question ultimately turned on the disputed issue of the secretary’s true reasons for adding the question late in the process of census preparation (Department of Commerce v. New York, 2019). It is noteworthy that the trial record and parts of the justices’ four separate opinions repeatedly allude to the statistical community’s concerns about accuracy and completeness.

In the specific instance of the citizenship question, there were concerns that immigrant families, resident in the United States both with and without documentation, would avoid the census altogether—presenting a problem of completeness—or would answer this question incorrectly or break off their answers at this question—presenting problems of accuracy. Observers speculate that the recent, well-publicized political issues such as the Border Wall, the impasse with asylum seekers at the southern border, the detention and separation of families, the attempted ban on entries from some Muslim countries, and intensified enforcement by ICE (U.S. Immigration and Customs Enforcement) would particularly chill the willingness of immigrants and their families to cooperate with the census (Levitt, 2019).

The Constitution requires an “actual enumeration” of the population, and it specifies one exclusion, “Indians not taxed,” which suggests that other exclusions could be added but were not. For over 200 years the census has counted inhabitants who could not vote: children, the incarcerated, immigrants, the enslaved, and, until the 20th century, women. The addition of the census question, however, would allow sorting on the basis of citizenship.

The citizenship question was last asked of all households in 1950, and it was a subquestion for those whose place of birth was outside the United States. It appears that after that date the Census Bureau viewed the statutory requirements of the Immigration and Nationality Act of 1952 (Pub.L. 82–414, 66 Stat. 163, enacted June 27, 1952, and called the McCarran-Walter Act) to suffice as a register of noncitizens.^5^ Since that registration requirement was abolished, citizenship has been asked on various surveys and in some administrative forms; the Department of Commerce told the Supreme Court that the government believed it had citizenship data on 90% of the population, and that adding the question to the census could improve that coverage to perhaps 93% of the population. There remained uncertainty about effects of the census, including correctly estimating any increase in undercount and any increase in response error from households who skipped the question or answered it untruthfully. Personnel at the Census Bureau invested a good deal of time and effort into estimating the probable effects of adding the citizenship question. Several analyses and one experiment suggested that the question would lead to an increase in undercount and data inaccuracy (Baum, Dietrich, Goldstein, & Sen, 2019; Brown, Heggeness, Dorinski, Warren, & Yi, 2018, 2019; O’Hare, 2018a; Velkoff, 2019).

## 7. Is There an Alternative to the Census?

On July 11, 2019, President Trump called for all agencies to provide their existing citizenship data to the Department of Commerce and announced that this step would produce a superior result to adding the citizenship question to the census. Setting aside the issue of the obvious contradiction between what the government told the Supreme Court and what it was now telling the public, it will require considerable statistical proficiency to merge the relevant databases to estimate a count of noncitizens with their geographic distribution.

The entire episode raises the issue of whether the census is really needed because alternative data bases are just as good. This issue presupposes that some other database or concatenation of databases is equally complete and accurate. It is perhaps apocryphal, but for years I have heard the story of the congressman who in Census Bureau oversight hearings held up the *Statistical Abstract of the United States* saying, “We don’t need a census because we have this book.” (See Anderson, 2015b, for a similar anecdote.)

Sometimes it is argued that we have enough sample surveys to substitute for a census. There are many sample surveys, but they require a sampling frame, which is ultimately derived from the census.

Sometimes it is argued that the post office could conduct the census. The post office is heavily engaged in the canvassing effort to locate addresses and it performs other services related to the census. Even with its great number of addresses, however, the post office does not comprehensively cover the population. Post office boxes complicate the geographic mapping of persons to physical addresses, and knowing a mailing address by itself offers no information on the size or composition of a household (GAO, 2017; Plevins, 2019).

One alternative to the census, the continuous population register, works in some countries, such as those in Scandinavia (Poston & Bouvier, 2017, pp. 46–54). The population register, which has its European origins in Catholic and Lutheran parish records before modernity, tracks births, deaths, and residence on a continuous basis for the entire population (Shryock, Siegel et al., 1976, pp. 13) Sometimes other vital events such as marriages and divorces are also included. The Chinese tradition of registering population dates back to the Han dynasty and was adopted elsewhere in Asia (Taeuber, 1959, p. 261). Population registers need to account for migration, however, and the United States is a very mobile society. Americans are used to the idea of registering births and deaths with their states, but a requirement to register at the police station after moving would not sit well with many Americans and their mobile lifestyles.

The United States has many administrative records that might be at least partial population registers. Births and deaths are recorded by vital statistics offices. Social Security, especially Medicare, is believed to have nearly universal coverage of the senior citizen population. Should Medicare-for-all become a public policy, then there is a possibility that as a register it could eventually replace the census. Other partial registers include REAL ID drivers’ licenses, tax records, Selective Service (for men over 18 years of age), motor vehicle registries, and voting rolls. Credit agencies, although private, cover a large fraction of the population. For the time being, there is a constitutional issue to require the census, but in addition, use of these partial registers beyond their originally intended purpose will raise many of the same issues of privacy, confidentiality, and security that the census faces.

## 8. Can and Will the Government Keep the Census Data Confidential?

Embedded in the citizenship discussion but also in every discussion of the census is the privacy concern: the worry that census data on individuals would be shared with law enforcement, such as ICE. There are substantial legal protections of individual privacy (Privacy Act of 1974; Title 26 of the U.S. Code) and penalties for employees and contractors who violate the prohibitions (Title 13 of the U.S. Code), but these might not mollify the conspiratorial. The cybersecurity concern is multipronged, but one aspect of it is the fear that the federal government is not agile enough and skilled enough to prevent hacking of census data.

There are Americans who fear that their information will not be kept private and it will be shared with other agencies. The specific concern is that law enforcement (such as ICE) would requisition and receive information on individuals and their location from the census. Unauthorized immigration is not the only reason that people might fear the government. Tax evaders, persons living in illegal housing, fugitives from criminal or civil litigation, and people who fear stalkers or ex-spouses are a few examples of people who might be reluctant to complete the census returns.

For years it was believed as true, and I taught as true in my classes, that the Census Bureau did not reveal individuals’ information even under considerable duress to do so during World War II. As it turns out, historian Margo Anderson (2015a) and her colleague William Seltzer (Seltzer & Anderson, 2007) deduced that the bureau had in fact collaborated to help identify Japanese American citizens and Japanese resident aliens and their locations.^6^ Since 1947, the Census Bureau and all of its personnel are required by statute not to share any individually identifiable information and the bureau has repeatedly made strong public affirmations of confidentiality (Jarmin, 2018). The success of the 2020 census will depend in part on whether the members of the public believe the Census Bureau will not reveal their individual information.

Considerable effort is underway at the bureau to develop a technique called differential privacy to ensure that no one could reverse engineer publicly available census data to yield information on individuals (Abowd & Velkoff, 2019). The Census Bureau has for years been concerned about uniquely identifiable individuals. An example of privacy protection is the top-coding of income data to prevent identification of persons with unusually large incomes. The concern remains that a household from a small geographic area such as a census tract might be identifiable if it were known to have some characteristic that distinguished it from its neighbors, such as a distinctively large household or a racial identity different from the rest of the neighborhood.

More recent is the fear that one’s information will not be private because, despite all the best intentions of the Census Bureau, the data will be hacked. The federal government’s track record in cybersecurity is not reassuring. The massive security breach at the Office of Personnel Management (OPM) illustrates the concern (Fruhlinger, 2018). Millions of Americans learned that their fingerprints, date of birth, and other uniquely identifying information were presumably in the hands of a foreign power. If OPM could not keep the data safe, why could the Census Bureau?

The efforts by the bureau to make an online response mode available to all households has intensified the concern that a malicious actor could provide disinformation, hack what individuals receive from the Census Bureau, or perhaps hack completed forms they submit to the Census Bureau (Wang, 2019). Suppose, for example, that a malicious power was able to infiltrate the cyberinfrastructure of the census and systematically edit certain variables—perhaps race or ethnicity—to change the apparent character of a neighborhood or a city. Suppose further that these efforts were aimed specifically at electorally purple areas, perhaps to encourage more election spending there. Alternatively, suppose that this same bot could delete households from the count entirely, potentially affecting representation and distribution of federal benefits and in the process sowing confusion and discord. The Census Bureau has announced a partnership with Microsoft and plans to harden and encrypt census data, and they have begun to publicize these developments (U.S. Census, “Security, Intrusion, and Detection,” n.d.). Perhaps their publicized attention to the perceived threats will reassure some of the skeptical public.

## 9. What About All the Errors Already in the Data?

Skepticism about any data is a healthy practice, at least to the extent that it is accompanied by empirical investigation. Few people are initially more skeptical of new data, nor more willing to apply multiple tests to the data, than statisticians. They examine their tests of the data to establish how trustworthy the data are.

My dissertation supervisor at the University of Chicago, Philip M. Hauser, was for a while the acting director of the 1950 census. When he was interviewed for his first job at the Census Bureau in the 1930s, he told the director his belief that the bureau would do well to reduce the undercount in subsequent censuses. Challenged to prove that there had been an undercount, Hauser purportedly took volumes of the 1930 and 1920 censuses to show that more people aged 10–19 had been enumerated in 1930 than the number aged 0–9 who had been counted in 1920. By now it is well known that children, especially babies, are often undercounted, and so are young families moving intrastate or interstate. In years of low immigration such as the United States after 1924, an undercount was the most reasonable explanation for the discrepancy.

We know about errors of enumeration and response precisely because government statisticians at the Census Bureau and other agencies have been relentless is examining census data. This practice leads to better understanding of the growth and characteristics of the population, and it leads to improved techniques for future efforts. In general, one would think that such efforts are reassuring as evidence of the intention to get closer to the truth, but sometimes the discussion of results may further complicate public mistrust.

Consider the efforts to portray the data with statistical rigor: where a statistician might see a statistic appropriately bounded by uncertainty—and in that sense, ‘true’—someone else might see fake news or an alternative fact. Statisticians may see sampling and estimation as legitimate efforts to get closer to ‘the truth,’ and thus to a more accurate count. In a rhetorical twist, statisticians’ professional concerns with error, uncertainty, and the treatment of missing data can be turned against the census truth claims to reinforce the idea that census personnel are ‘making it all up.’

Sometimes the request is to use such methods to correct an undercount for missing data or sometimes for entire households. Whether this editing violates the “actual enumeration” mandate has been a matter for judicial review. The constitutional arguments are briefly reviewed at UpCounsel (n.d.).

Given that there are undercounts, sometimes there are demands for editing. Some mayors and governors have claimed that a differential undercount was shortchanging their geographic area in terms of representation and funding (Capps, 2018; Pratt, 2019). Leaders of various interest groups may also take up the cause that their constituencies have been overlooked. Such claims are not surprising, given the high stakes of enumeration, but they are also well publicized. Ironically, the errors that are documented by careful statisticians may end up being used against the bureau amid claims of favoritism, partisanship, or incompetence.

## 10. Errors of Coverage and Accuracy Reinforce Mistrust of Government

There are many reasons for census errors. Errors of coverage—entirely missing entire households or missing individuals within households—may come about quite innocently, because Americans are highly mobile and they might somehow miss all the publicity around an enumeration.^7^ There are hard to count populations, such as the homeless, people living in rural areas, movers, and those living in new and unlisted housing. And as mentioned above there are also people who don’t want to be counted due to fear.

It does seem likely, however, that the classic sort of enumeration resisters is still with us: those who don’t trust the government in general or the Census Bureau in particular, whether their motivations be religious, ideological, economic, or social. The census is required by law, but the Census Bureau uses prosecution only as a last resort to secure compliance (Mabeus, 2014). Realistically, individuals who wish to avoid the census will also avoid prosecution. They may receive many follow-up calls, but there have been no prosecutions since the 1970 census.

The societal effect of nonparticipation or partial participation, such as failing to list some members of a household, is an error of coverage (National Research Council, 2007). People may also refuse to answer particular questions; this is response error. Without the long form that was formerly used, the coming enumerations should escape the long-standing complaints about housing questions, income questions, and other questions deemed ‘too intrusive.’ Even as simple a question as age, however, can have response error from the ignorant, careless, or vain.

Undercounts, especially of a minority population, are of immense interest (O’Hare, 2018b). In the political back-and-forth around discussion of undercounts, it is easy for the errors to be attributed to the Census Bureau, their procedures, and their personnel. These issues will not abate in 2020. The long-term population decline in the Northern tier states and the continued growth of the Sunbelt will cause painful realignments in Congress. There are always deep, varied interests in claiming that the count is too low and that important groups have been omitted, and that a postcensal adjustment is needed.^8^

To its credit, the bureau reaches out repeatedly to local governments and community leaders for assistance in improving coverage and accuracy. The current Complete Count Committees represent a widespread proactive effort to ensure that the 2020 census is complete (U.S. Census, “Complete Count Committees,” n.d.). These local groups assist both the bureau and their communities in a variety of ways, including providing addresses of new housing, assisting with information about counting the local migrant or homeless population, and publicizing the aims of the census to the community. Advisory committees and groups of various sorts provide the Census Bureau with information. Nevertheless, in a very large and varied country, dissident voices will inevitably be raised.

It is appropriate for statisticians, demographers, and others to assess errors in the data. Statisticians strive to be careful and precise in their data presentations. They test their data in multiple ways. It is customary to report standard errors, for example. Techniques that are uncontroversial within the professional community may arouse suspicion among some of the general public. What is smoothing? How about imputation? Exactly what do you mean by differential privacy? For a population that might not be statistically literate, it may be easy for a commentator to posture by saying that ‘no one really knows’ or ‘they have made it all up.’

The worst outcome here would be that the existence of an undercount, the treatment of missing data, the analysis of response error, and other routine and legitimate data treatments lead to labeling the census fake news, and the Census Bureau personnel just another manifestation of the deep state.

## 11. Neither Checks nor Balances Preserve the Integrity of the Census

The census is a large, complex undertaking, requiring huge expenditures of time and money and vast efforts to tabulate and analyze. Congress needs to appropriate the funds and the executive branch needs to be effective and efficient in carrying out the census. Bringing about a successful census requires that all parts of government cooperate.

The courts have generally shown deference to the professionalism of the Census Bureau, but there have been increasing judicial efforts to affect how the census is carried out.

The recent Supreme Court decisions over gerrymandering and the citizenship question, issued on successive days in June, have interesting implications for each other (Department of Commerce v. New York, 2019; Rucho v. Common Cause, 2019). In a nutshell, the gerrymandering decision turned on the issue of whether legislatures could develop voting districts in such a way as to maintain a majority of voters from the dominant party and therefore maintain their power in the state legislature for a decade. In a split decision, the Supreme Court denied that the courts had a role to play in a purely political decision such as redistricting (Rucho v. Common Cause, 2019).

This is important for the citizenship question decision, issued the next day, because in that case the Court held that the secretary of commerce had violated the Administrative Procedure Act of 1946 (5 U.S.C. §500 et seq.) by offering a pretextual reason for including the citizenship question: the need to enforce the Voting Rights Act of 1965 (52 U.S.C. §10101). If the secretary had been more forthcoming with his real reasoning, the opinion suggested, then there would be no judicial impediment to including the citizenship question (Department of Commerce v. New York, 2019).

Political observers believed that the secretary’s real reason was this: if legislatures could redistrict using number of *citizens* instead of number of *inhabitants*, then districts could be more precisely drawn to benefit one party (Edmonston & Wines, 2019; Henderson, 2019). First, however, the state legislatures need data on citizens, not inhabitants. In short, if the secretary had given this reason, the Court’s decision on gerrymandering—that it had no role to play in a purely political process—would seem to imply that its decision on the citizenship question would have been different, and the citizenship question would have been included on the census forms in 2020. Arguably, even a nakedly partisan reason would be sufficient if the other requirements of the Administrative Procedure Act were met.

I want to draw a broader conclusion from this comparison, however. Jealousy over the prerogatives of one’s branch of government has often provided an effective check on the other two branches, even if all involved are from the same party. This result, however, would depend upon party discipline. If a single party controls all branches of government, and if it had enough discipline such that party affiliation was paramount, then there seem to be no *structural* checks or balances to protect the decennial enumeration from questions and practices that are unwise or will increase error. Today there is enabling legislation to preserve the integrity of the statistical system, but legislation can be repealed with large enough legislative majorities.^9^ What is true of the Bureau of the Census would also be true of all other statistical agencies. The Bureau of Labor Statistics experiences continuous political scrutiny for how unemployment data are collected and reported. Many other agencies—the Defense Department and Veterans Administration, the Department of Education, Health and Human Services, Homeland Security—would similarly be unprotected.

## 12. Statisticians With Integrity Are the Best Defenders of this Pillar of Democracy

This summary may present a bleak prospect, with statisticians the unwilling tools of politicians interested in power and not in truth. I am by no means so pessimistic as that sounds. The profession of statistics is healthy for democracy, and statisticians’ personal commitments to integrity are important safeguards for our statistical infrastructure (American Statistical Association, 2018). In an important way that politicians and others could learn from, this is a profession dedicated to telling the truth.

Moreover, the truth-telling takes place in a give-and-take in which conclusions may be challenged, analyses redone, and results argued. The value of this vigorous questing in the long run will outweigh any short-run concerns that there is data tampering or hiding of inconvenient findings.

One of the most reassuring aspects of the decennial census is the bureau’s openness about the robustness of the data, the effort to improve their methods through identifying sources of error, and the continued publication of results. Statistical innovations are always going to be challenged, and the first set of challenges will be issued from others within the community. But the larger enterprise in which those innovations arise—creating a better, more complete, more accurate set of data—is something all Americans should respect.

Because so much rests on the census and the rest of the social statistical infrastructure, statisticians are integral to democracy. And while most statisticians are too modest to view themselves as heroes, their work on Census 2020 preserves an important democratic institution and helps assure the quality of the larger social statistical infrastructure.

## Footnotes

1. Through the 1860 Census, enslaved persons were enumerated, but counted for representation purposes as three-fifths of a person. The original wording of the Constitution allocated direct taxes by population, later eliminated in the wording of the Fourteenth Amendment. Two concerns motivated the three-fifths compromise: both the Southern states and the Northern states wanted more representation, but they also wanted to shift more of the taxation burden to the other region. The three-fifths figure was a compromise between the Northern states and the Southern states. Apportionment proved unsuccessful for taxation because wealth was not distributed in the same way as the population (Johnson,1998, 2004, 2019). The three-fifths ratio was eliminated by the post-Civil War Fourteenth Amendment. See Anderson (2015b, pp. 11-13 et seq.).

2. Compare the divinely ordained census in Numbers 1:12 with the punishment resulting from the unauthorized census in 2 Samuel 24; also see 1 Chronicles 23. On the controversy, see Anderson (2015b, pp. 9).

3. By local lore, Jefferson also helped enumerate Albemarle County, Virginia, but this belief might have come from the fact that his name was added in the margin of the return. Compare his known signatures with the image of the enumeration page from the 1790 Censes, which appears consistent with his other signatures but inconsistent with the hand in which the other entries are written. Returns from the 1790 Census had to be publicly posted for correction, and one possibility is that rather than being the enumerator, Jefferson was merely adding himself to the count.

4. The The Pew Research Center (2019) reports that trust in government is at historic lows in the United States. Secretary of Commerce Wilbur Ross mentioned mistrust of the census specifically in remarks to Congress (Gunter, 2017).

5. It is not clear how complete the alien registration was, but the Census Bureau argued that asking citizenship of the entire population “had become of less importance compared with other possible questions to be included in the census, particularly in view of the recent statutory requirement for annual alien registration which could provide the Immigration and Naturalization Service, the principal user of such data, with the information it needed.” (U.S. Bureau of the Census, 1966).

6. Census data have not always been confidential. In the 1790 Census, the returns were posted in two locations in every county to permit residents to make corrections. Over time, however, the expectation of confidentiality of individual returns grew strong (U.S. Census Bureau, “Events in the Chronological Development,” n.d.). A change in wartime legislation had made this data sharing legal. Gatewood (2001, pp. 14–16) documents the variety of statements from the bureau between 1988 and 2000 about this episode. Minkel’s (2007) article cites Margo Anderson and William Seltzer’s discovery in 2000 of evidence to document the data sharing. Minkel opens with the sentence “Despite decades of denials, government records confirm that the U.S. Census Bureau provided … names and addresses of Japanese-Americans …” See also Aratani (2018); Asian Americans Advancing Justice (n.d.).

7. A reviewer of this article has helpfully noted that there are also errors that come about through erroneous inclusion—double-counts, counts of children born after census day, and so on. The issue of public mistrust, however, has centered on undercounts.

8. Efforts to require such adjustments in the apportionment counts have so far not survived judicial scrutiny.

9. Title 13 of the U.S. Code provides for the administration of the census, collection and publication of statistics, offenses and penalties, and exchange of data with other agencies, states, and localities. There is protection for other data, such as tax data. See Internal Revenue Code (2018, 2019).
